# A Treatise on the Formation, Constituents, and Extraction of the Urinary Calculus; Being the Essay for Which the Jacksonian Prize for the Year 1833 Was Awarded by the Royal College of Surgeons in London

**Published:** 1846-02

**Authors:** 


### BIBLIOGRAPHICAL NOTICES.

*A Treatise on the Formation, Constituents, and Extraction of the Urinary Calculus; being the Essay for which the Jacksonian Prize for the year* 1833 *was awarded by the Royal College of Surgeons in London.* By JOHN GREEN CROSSE, M. D., F. R. S, Senior Surgeon to the Norfolk and Norwich Hospital, Lecturer on Clinical Surgery, Member of the Royal College of Surgeons, and Fellow of the Royal Medical and Chirurgical Society of London, &c. &c. 1 vol. 4to. pp. 231. London: John Churchhill. 1841.

This work of Mr. Crosse has not yet been republished in this country, though, from the essentially practical nature of the subject of which it treats, avoiding all theoretical speculations, we trust that an American edition may soon be laid before the profession.

The work is divided into twelve chapters, and contains a very clear and concise account of the causes, composition, and growth of calculi in the kidneys, ureters, bladder, prostate gland and urethra; the mode of sounding for stone, the removal of it when found to exist, and the after treatment—the whole occupying ninety-five pages. We have, then, twenty-nine plates, showing calculi after their removal from the bladder, and also in the various situations in which they are found in the body, together with engravings of the different instruments used in their extraction.

The volume also has an appendix of 22 cases of "*lithocystotomy*," and 704 cases, occurring chiefly in the Norfolk and Norwich Hospital, with their results; together with an alphabetical catalogue of the express treatises upon gravel, stone, and lithotomy, published in different ages and countries, and of essays or notices referring to those subjects in many periodical works, occupying sixty-five pages.

Dyspepsia is stated by Dr. Crosse to be the principal constitutional cause of calculus, combined with want of exercise, variable climate, peculiar diathesis, or some local disease in the urinary apparatus; the use of spirituous liquors or acids, which quickly cause an appearance of lithic acid, is also considered a powerful predisposing cause of this disease; alkalies act also in the same way, by creating alkaline deposits.

The sudden vicissitudes in temperature, so frequent in the temperate zone, by throwing suddenly a much greater degree of exercise on the functions of the kidneys, while the same effect is not produced either in hot climates, or in the very cold ones, is also considered a very strong exciting cause of the formation of calculi.

The local causes are blows upon the loins, injuring the kidneys, or any cause which may prevent the free excretion of urine, as enlarged prostate, strictures, &c., which act not only by the inflammation excited, but in the last mentioned cases by preventing the passage of small stones, that might have been readily voided under other circumstances.

Hernia of the bladder, sacculi formed by the mucous membrane passing between the muscular coat, and forming pouches for the lodgement of the urine, by causing inflammation of this organ also predisposes to this affection, though their existence is by no means so frequent as the other causes which are mentioned.

Extraneous substances in the bladder are also mentioned as forming nuclei for stone ; a fact which is well known to any one at all acquainted with this subject.

On the subject of the chemical composition of calculi, the author insists upon the importance of paying great attention to the nuclei, as the analysis bears so directly upon the prevention of the disease. Of 100 cases of calculi, passed per urethram, 72 were found to consist of lithic acid, or lithate of ammonia, and 14 of the oxalate of lime.

These last mentioned calculi grow much more slowly than those composed of the phosphates; but all calculi, as a general rule, continue to increase as long as they remain in the bladder; so that, by comparing the size of a calculus, on sounding, with the length of time it has been forming, as indicated by the symptoms, some idea may be formed of its density and composition. This, however, must be very vague, and it requires long experience on the part of the surgeon to be able to pronounce, with any certainty, on the size and composition of a stone; and more information is derived from the *tactus eruditus* in sounding, than from anything else.

The author does not think that calculi are often adherent to the bladder; they may be fixed by extending into the ureters, or urethra, or grasped by the muscular fibres of the bladder, or contained partially in a sacculus; but, except by a layer of lymph soft enough to be easily broken through, his large experience has brought him *no instance whatever* of a calculus actually adherent to the coats of the bladder,—a very important point to remember, particularly with regard to the operation for lithotripsy.

With regard to calculi in the kidneys, less danger arises from large than small ones, as, in the first case, the organ may become atrophied, and give rise to comparatively little inconvenience, unless both kidneys are involved, when, of course, the case must terminate fatally in a few days; but a smaller calculus, suddenly blocking up the ureter, may cause fatal suppression of urine, in case the organ of the other side should have been previously incapacitated from disease, or by inducing an attack of acute nflammation in one kidney, which may involve its fellow. Calculi fixed in the commencement of the urethra may grow to a considerable size, occupying the prostatic portion of the urethra, and extending upwards into the bladder. The presence of a stone here is always attended with great suffering, and usually accompanied with a constant stillicidium.

The urine not remaining in the bladder, this organ at length becomes so contracted that there may not be room enough in the viscus to receive the end of the sound. It is highly important to be aware of this state of things; and much light may be thrown on the case by an examination per anum, and by the stone being felt by the sound before it has passed deep enough to have entered the bladder, in addition to the ordinary signs of calculus.

The plan proposed by some of removing the calculus, when in this situation, by an incision just within the sphincter ani, is not recommended by the author; but he prefers the usual lateral incision through the perineum, or when the staff cannot be introduced into the bladder, cutting upon the *gripe*, by which the danger of a recto-vesical fistula is prevented. Under these circumstances, we are cautioned not to be led into error by mistaking a small stone, which can be touched by the sound in all directions, for a large one; and the staff should be introduced on the pubic side of the stone, and not posteriorly, for fear of wounding the rectum. Cases are mentioned in which a stone, not extending into the bladder, remained stationary for years, with little inconvenience, though, when complicated with stricture especially, the very reverse is often the case.

Large calculi, occupying the membranous part of the urethra, occasionally cause suppuration, and a discharge through the perineum, without any operation, or they may descend into the scrotum, and occasionally increase to an immense size, by the opening through which they passed remaining open, so that the urine gets access to them.

Our attention is drawn to those calculi which have passed down from the kidneys, and lodged in the prostatic portion of the urethra, and the true prostatic calculi, which are formed in the ducts of this gland, and are uniformly composed of phosphate of lime. These last give rise to none of the characteristic symptoms of stone, unless they project at the orifices of the prostatic ducts, or escape into the urethra, or when the concretion is large, or numerous small calculi, collected into a cyst, press upon the urethra.

Stricture, or any other disease causing inflammation of the prostatic portion of the urethra, and interrupting the free exit of the excretion of the prostatic fluid, are considered the causes predisposing to the formation of calculi in this situation. Another mode in which concretions are formed is also noticed, usually occurring in old persons—where we have hypertrophy of the prostate, and a diseased condition of the bladder—and that is, deposits of phosphate of lime in the varicose veins around the neck of the bladder, from the size of a pin's head to that of a kidney bean. These deposits have no connection whatever with urinary concretions, but are a morbid growth from the outer coats of the veins, to which they always adhere when small, and are covered by a membrane, which is the extended inner coat of the vessel.

The symptoms of stone in the bladder are passed over; but we have some valuable remarks on the pathological changes produced in this organ by their presence.

We next come to the operation of sounding for a stone, than which, as too truly remarked by our author, "there are few operations more abused and less skilfully practised." There are many morbid conditions of the bladder, giving rise to symptoms of stone, and, by the persevering efforts of the surgeon to find what may not exist, cystitis and death may be produced. This is especially the case when tumours exist in the bladder; and the symptoms here resemble very much those of stone. The bladder is one of the seats of polypi, of a benign nature, even in children; and the surgeon, under these circumstances, is very liable to be led into error. The immense importance of sounding is insisted upon, and the frequent cases of this kind, in which cystotomy has been performed, even by the most experienced surgeons, are mentioned.

Vesical auscultation is also regarded by our author as a valuable adjuvant in doubtful cases.

We next have a chapter on removing small stones from the bladder by the urethro-vesical forceps of Weiss, and many important directions for the performance of this operation, which may often save the patient from the hazard of lithotomy.

On the important subject of lithotripsy Dr. Crosse says but little, owing, as he remarks, to his want of experience on this subject, and most truly remarks, that so much delicacy and tact are required for the safe employment of instruments on the living subject, that they are scarcely employed by any, save those who dedicate their time and attention almost exclusively to the undertaking.

Since this work was written, however, the repeated and brilliant success met with in this department of surgery has been so great, that we may reasonably hope, before long, to see the time when lithotomy, in the *adult*, will be the exception to the general rule; but the great error has been committed of regarding lithotripsy as an easy operation, and one requiring but little study, and hence the small degree of favour with which this mode of removing calculi has been regarded by many who have undertaken its performance, without possessing qualifications necessary to ensure success.

In speaking of the removal of the stone from the bladder by the cutting operation, the author proposes the term Litho-cystotomy in preference to lithotomy. The operation above the pubes, and by the rectum, are justly regarded as applicable to but few cases, and the directions given have reference entirely to the lateral operation through the perineum, in two ways; first, with the curved, and secondly, with the straight staff. The importance of forming some idea of the size of the stone to be removed is insisted upon, not so much from its great size being an obstacle—as the author mentions a case in which a stone weighing fourteen ounces was successfully removed, and the patient lived five years afterwards—but because this dangerous operation may be, and has been resorted to, where the calculus was so small that it might have been readily removed by the urethro-vesical forceps; and our author states, that he has repeatedly known the stone so small as to pass through the wound with the urine, as soon as the bladder was opened, and escape detection; and sometimes, where the operation has been prolonged in a tedious search for the stone, chiefly owing to its small size, it has proved to be such as might readily have been brought away by the urethro-vesical forceps.

The directions for the operation are such as are usually given, until the third stage of cutting into the bladder with the scalpel—not with the gorget, as usually practised in this city. Dr. Crosse prefers a staff, with a deep semi-circular groove upon the whole of its convex and the adjoining portion of its straight part; and, after opening the membranous part of the urethra, the knife is passed on in the groove of the staff, supported by the forefinger of the left hand, and the incision can be enlarged as far as necessary in the withdrawal of the knife. This stage of the operation, he remarks, he has "found, from ample experience, very difficult to execute upon the *curved staff*, owing to its being held obliquely, to present the groove favourably, and its receding from you in two directions."

The straight staff is calculated to obviate many of these difficulties, and possesses strong recommendations, in our author's opinion, for a preference; and, as it is a novelty to most operators in this country, where the gorget is usually used, we extract the whole account. It should be premised that the staff is to have two handles, one at right angles to the other.

"After executing the first and second stages, as already described, the second handle is turned towards the right side, in order that the groove of the staff may be presented in the opposite direction; in doing this, you find in the straight staff the great advantage of the instrument moving round its axis, and its position in the urethra consequently not altering from the median line, which I deem a very essential point. The straight staff, in passing through the membranous part of the urethra, lifts it up from the rectum, pressing against the pubic or superior surface of the passage, thus affording great protection against wounding the rectum: the reverse happens with the curved staff, its convexity pressing towards the rectum, and rendering it not easy always to avoid wounding it. The greatest gain from the straight staff is in the facility given to the third stage of cutting into the bladder, by the instrument answering to the median line at the same time that the groove is presented in the most favourable position, and by your having to cut in a straight direction—so that, getting down to the staff, you find this third stage converted into one plain continued incision, effected by carrying on the knife in the groove, as you would carry it along a common director, till satisfied that you have gone as deep as required, passing the prostate, and just entering the bladder; you then enlarge the incision in withdrawing the scalpel."

The cutting gorget is rejected *in toto*, as, in the hands of the generality of operators, it is found to bring great peril, where any error occurs, such as its passing between the bladder and rectum, or between the former and the pubes, instead of entering the bladder, or, after entering it, transfixing its coats from within outwards; all of which accidents he has known to happen, and all of which are necessarily fatal in the adult, except where the injury is towards the rectum.

In using the curved staff, the blunt-beaked gorget is recommended, after completing the section into the bladder, and then the staff is to be withdrawn, and the gorget is used as a conductor, and also as a dilator of the neck of the bladder. When the straight staff is used, it is withdrawn before introducing the finger into the wound.

The extraction of the stone is justly regarded as the part of the operation requiring the most feeling and judgment, and the greatest care is to be taken not to use too much force, for fear of inducing inflammation and sloughing. Pouteau's directions are quoted, inculcating the immense importance of *slowness*, *gentleness*, and *patience*, by which very large stones can be extracted without danger; and if much resistance is met with, the wound is to be enlarged to the requisite extent, by which we are sure to have a safe, though, perhaps, not a rapid and brilliant operation. The much greater facility with which the neck of the bladder dilates in children than in adults is also to be borne in mind.

The after treatment is such as is usually recommended; and we then have a chapter on hemorrhage after the operation—one of the most embarrassing accidents to the surgeon, and one attended with great danger to the patient, both from its immediate and remote effects. In old persons we have venous hemorrhage, occasionally to an alarming, and sometimes fatal extent, from the varicose veins around the bladder and prostate gland, which are divided in the operation. The plan adopted by Dr. Physick, of introducing a catheter into the bladder, and inserting a strip of lint between the edges of the wound, will here be found the most effectual treatment.

The arteries entering the corpus spongiosum at the bulb are often divided, and give rise to severe hemorrhage; the wounding of the corpus spongiosum itself seldom furnishes an alarming bleeding; but when the arterial branch, before entering the bulb, is cut, we may expect very different results. When this occurs, and the vessel can be secured at the place of division, it is of course to be practised, or, if we cannot secure it at this point, we may control it by keeping up pressure on the internal pudic, or, by securing this vessel, on the ramus of the ischium. When plugging the wound is necessary, in consequence of not being able to secure the vessel in any other way, the operator may, with good reason, apprehend some untoward symptoms. The division of the pudic artery itself, a rare occurrence by the way, of course requires the ligature, which can be applied by a small curved needle in the forceps. If hemorrhage does not occur within a few hours, we seldom have any cause for anxiety from this source, although, among the old writers on lithotomy, it was by no means uncommon to have secondary bleeding coming on a week or two after the operation, proving the violence done to be so great as to have caused sloughing.

Of the plates we say nothing—because they must be seen to be properly understood—except to speak of one, in which there was such immense enlargement of the ureter, following stricture and four small calculi in the bladder, that it looked like an intestine. A similar case came under our own notice some years ago, where the ureter was mistaken for the extremity of the colon and tied, under the impression that it was the gut, before the mistake was discovered. In this case an impassable stricture had existed for some years, and the patient suffered from an incurable fistula in perineo.

From the table of 704 cases of lithocystotomy, performed at the Norwich and Norfolk Hospital, we find the average whole number of fatal cases to be 1 in $7\frac{53}{93}$; and of these, 669 were males, in whom the proportion was 1 in $7\frac{32}{91}$; and of the remaining 35, who were females, but two died, being 1 in $17\frac{1}{2}$; 262 were children under 10 years of age, in whom the proportion of fatal cases was 1 in $14\frac{15}{19}$; showing the truth of the opinion, that this operation is far more dangerous in adults.

The catalogue of express treatises upon stone, &c., which concludes the work before us, is very large, and will afford valuable assistance to any who may wish to review the whole history of this interesting department of surgery.

In the foregoing remarks and extracts, it has been our aim rather to give a condensed view of the information to be derived from the work before us, on a subject that is now attracting even more attention than usual, in consequence of the great improvements introduced within a few years, than a review; and we again repeat, that we hope, before long, to see the entire work laid before our readers in an American edition.

